# The Kainate Receptor Subunit GluK2 Interacts With KCC2 to Promote Maturation of Dendritic Spines

**DOI:** 10.3389/fncel.2020.00252

**Published:** 2020-08-26

**Authors:** Sebnem Kesaf, Stanislav Khirug, Emilie Dinh, Marta Saez Garcia, Shetal Soni, Ester Orav, Eric Delpire, Tomi Taira, Sari E. Lauri, Claudio Rivera

**Affiliations:** ^1^HiLIFE Neuroscience Center, University of Helsinki, Helsinki, Finland; ^2^Molecular and Integrative Biosciences Research Program, University of Helsinki, Helsinki, Finland; ^3^Developmental Biology Institute of Marseille, Marseille, France; ^4^Department of Anesthesiology, Vanderbilt University School of Medicine, Nashville, TN, United States; ^5^Veterinary Biosciences, University of Helsinki, Helsinki, Finland; ^6^Institut de Neurobiologie de la Méditerranée (INMED) UMR901, Marseille, France

**Keywords:** developmental delay, glutamatergic synapses, chloride homeostasis, actin cytoskeleton, synaptic plasticity

## Abstract

Kainate receptors (KAR) play a crucial role in the plasticity and functional maturation of glutamatergic synapses. However, how they regulate structural plasticity of dendritic spines is not known. The GluK2 subunit was recently shown to coexist in a functional complex with the neuronal K-Cl cotransporter KCC2. Apart from having a crucial role in the maturation of GABAergic transmission, KCC2 has a morphogenic role in the maturation of dendritic spines. Here, we show that *in vivo* local inactivation of GluK2 expression in CA3 hippocampal neurons induces altered morphology of dendritic spines and reduction in mEPSC frequency. GluK2 deficiency also resulted in a strong change in the subcellular distribution of KCC2 as well as a smaller somatodendritic gradient in the reversal potential of GABA_A_. Strikingly, the aberrant morphology of dendritic spines in GluK2-deficient CA3 pyramidal neurons was restored by overexpression of KCC2. GluK2 silencing in hippocampal neurons significantly reduced the expression of 4.1N and functional form of the actin filament severing protein cofilin. Consistently, assessment of actin dynamics using fluorescence recovery after photobleaching (FRAP) of β-actin showed a significant increase in the stability of F-actin filaments in dendritic spines. In conclusion, our results demonstrate that GluK2-KCC2 interaction plays an important role in the structural maturation of dendritic spines. This also provides novel insights into the connection between KAR dysfunction, structural plasticity, and developmental disorders.

## Introduction

The interplay between glutamatergic and GABAergic transmission is crucial for refinement and maturation of synaptic connectivity in developing networks. Impairments in the balance of neurotransmission may result in various neurodevelopmental and psychiatric disorders, caused by persistent changes in neuronal networks during development. K^+^-Cl^−^ cotransporter (KCC2) maintains a low level of intraneuronal chloride and hyperpolarizing GABA_A_ mediated inhibition in adult neurons (Rivera et al., 1999; Blaesse et al., 2009; Kaila et al., 2014). Also, we have previously shown that independent of its Cl^−^ extruding capacity KCC2 regulates the morphology of dendritic spines, which are specialized subcellular structures hosting glutamatergic synapses (Li et al., 2007). These two facets of KCC2 may allow synchronized regulation of both GABAergic and glutamatergic transmission and highlight KCC2 as an important therapeutic target for the regulation of pathological neuronal networks. Indeed, recent results show that perturbed regulation of KCC2 expression may be involved in the etiology of neurodevelopmental disorders (Tang et al., 2016, 2019; Hinz et al., 2019).

KCC2 interacts with the GluK2 subunit of kainate type of glutamate receptors (KARs), which play important roles in both pre and postsynaptic modulation and influence the functional development of limbic networks (Pinheiro and Mulle, 2008; Contractor et al., 2011; Lerma and Marques, 2013; Xu et al., 2017). The interaction with GluK2 regulates trafficking and surface expression of KCC2 in hippocampal neurons (Mahadevan et al., 2014; Pressey et al., 2017). Consequently, the efficiency of chloride extrusion at the plasma membrane that sets the reversal potential of chloride permeable GABA_A_ receptor is affected by the interaction with GluK2. The role of GluK2-KCC2 interaction for the morphogenic actions of KCC2 in dendrites on the other hand remains unclear. Although the GluK2 subunit containing KARs are implicated in the regulation of dendritic spines (Lanore et al., 2012; Petrovic et al., 2017), detailed mechanistic information on the role of endogenous GluK2 in spine maturation is lacking. Interestingly, the morphogenic role of KCC2 in the maturation of dendritic spines is mediated by the structural interaction with proteins regulating actin filament dynamics such as 4.1N (Li et al., 2007; Llano et al., 2015), which also interacts with GluK2 (Copits and Swanson, 2013).

In this study, we addressed the role of the interaction between the kainate receptor subunit GluK2 and KCC2 for dendritic spine maturation. We find that *in vivo* shRNA mediated gene silencing of GluK2 expression in CA3 hippocampal neurons induce altered morphology of dendritic spines and reduced frequency of mEPSCs. GluK2 deficiency also resulted in a strong change in the subcellular distribution of KCC2 as well as a smaller somatodendritic gradient in the reversal potential of GABA_A_. Strikingly, the aberrant morphology of dendritic spines in GluK2-deficient neurons was restored by overexpression of KCC2. GluK2 silencing in cultured hippocampal neurons significantly reduced the expression of 4.1N and functional state of the actin filament severing protein cofilin. Consistently, assessment of actin dynamics using fluorescence recovery after photobleaching (FRAP) of β-actin showed a significant increase in the stability of F-actin filaments in dendritic spines. In conclusion, our results demonstrate that GluK2-KCC2 interaction plays an important role in the maturation of dendritic spines. This provides novel insights into the link of KAR function to structural plasticity and its role in neurodevelopmental disorders. Our results suggest that rescue of dendritic spine morphology by KCC2 overexpression may be a contributing mechanism for the positive effect of KCC2 overexpression in different models of neurodevelopmental disorders (Contractor et al., 2015; Tang et al., 2016).

## Materials and Methods

### Experimental Animals

The experiments were approved by the Animal Care and Use Committee, University of Helsinki. The animals used in this study included wild-type, Thy1-EGFP [Jackson Stock Tg (Thy1-EGFP)], and KCC2^ flox/flox^ young adult mice of both sexes (Mavrovic et al., 2020).

Viral vectors GluK2 shRNA [GluK2 shRNA(2-2) pLKO.1/Syn1-EGFP] and the control (pLKO.1/Syn1-EGFP) were as described previously (Sakha et al., 2016) GluK2 shRNA (2-2) pLKO.1/Syn1-DsRED and the control pLKO.1/Syn1-DsRED viral vectors were constructed by replacing the EGFP fragment with DsRED. DsRED fragment was cloned from pLen-d-Syn1-DsRED using EcoRI and KpnI restriction sites. The constructs were verified by DNA sequencing. All lentiviral particles GluK2 shRNA(2-2) pLKO.1/Syn1-EGFP, Syn1-DsRED, the control pLKO.1/Syn1-EGFP and Syn1-DsRED were produced in HEK293FT cells as described in (Vesikansa et al., 2012). HEK293FT cells were seeded at the density of ~3 × 10^6^ on 10 cm^2^ Petri dishes and transfected with Fugene HD (Roche Applied Science) transfection reagent on the next day using 0.75 μg of envelope-coding plasmid pMD2.G; 2.25 μg of packaging plasmid psPAX2 and 3 μg of transfer vector. Medium containing viral particles (viral supernatant) was collected 48 h post-transfection and removed of cell debris by low-speed-centrifugation (at 3,000× *g* for 15 min) and concentrated with PEG-it™ virus precipitation solution (System Biosciences). The viral supernatant/PEG-it™ mixture was centrifuged at 1,500× *g* for 30 min at +4°C and the virus pellets were suspended in DMEM in 1/200 of the original volume. AAV8-CaMKIIa-mCherry-Cre (mCherry-Cre) and control virus AAV8-CaMKIIa-GFP (EGFP-control; UNC, Vector Core) was used for injections in KCC2^flox/flox^ mice.

### Stereotaxic Virus Injections

Between P10 and P12, all animals were anesthetized with Isoflurane (Isocare^®^ Vet, 1,000 mg/g, air flux at 240–250 ml/min, anesthesia induction at 4.8%, maintenance at 1, 5–3%) and received two bilateral injections in the CA3 region of the hippocampus (1st AP: −1.4 mm, ML: −1.7 mm, DV: −1.8 mm; 2nd AP: −1.9 mm, ML: −2.2 mm, DV: −2.1 mm; coordinates from Bregma). Anesthetized mice were placed at a stereotactic frame (World Precision Instruments, C1TO1-034), with their head fixed horizontally, and their body temperature maintained at 36°C degrees. A Hamilton syringe (World Precision Instruments, Nanofil Syringe, Lot: 01C) with a glass capillary filled with mineral oil was used for the virus injections. Prior injection, the capillary was backfilled with 2 μl of ready to use virus aliquots (5 μl, diluted half in glycerol and green dye) using a microsyringe pump [World Precision Instruments, Micro4™, MicroSyringe Pump Controller, 10 SNL (nl/s)]. Injection of the viruses (400 nl per injection site) was made at a constant speed of five SNL with an inter-injection interval of five min to ensure proper distribution of the virus. Finished the surgery, all animals were tattooed in the paws for identification and placed back to their home cage for recovery.

### Whole-Cell Patch-Clamp Gradient Recordings

The animals were anesthetized, and 350 μm brain slices were cut using a Vibratome 3000 (Vibratome). Slices were bathed in a standard physiological solution containing the following (in mM): 124 NaCl, 3 KCl, 2CaCl_2_, 25 NaHCO_3_, 1.1 NaH_2_PO_4_, 2 MgSO_4_, and 10 D-glucose, equilibrated with 95% O_2_ and 5% CO_2_, pH 7.4 at the experimental temperature of 32°C. The slices were allowed to recover at 36°C for 1 h before the experiments were started. The composition of the patch pipette solution was the following (in mM): 18 KCl, 111 K-gluconate, 0.5 CaCl_2_, 2 NaOH, 10 glucose, 10 HEPES, 2 Mg-ATP, and 5 BAPTA, pH was adjusted to 7.3 with KOH. The resistance of the patch pipettes was 6.5–7.5 MΩ. The membrane potential values were corrected for a calculated liquid junction potential of 10 mV (Barry, 1994).

To assess chloride homeostasis and the dynamics of chloride removal in CA3 neurons, we use an assay described by Khirug et al. (2005). Neurons were recorded under visual guidance in whole-cell patch-clamp mode, whereupon caged GABA was photolyzed with brief pulses from a UV laser (Pettit and Augustine, 2000; Khirug et al., 2005) along the dendrite at 50 μm from the soma of pyramidal neurons. To expose KCC2 to the Cl^−^ load at the soma, we voltage-clamped the neurons in whole-cell configuration with 19 mM Cl^−^ in the patch pipette. The values of E_GABA_ were obtained from I-V curves. Under these conditions, experimentally recorded somatic E_GABA_ is very close to the value calculated based on the Nernst equation (−50 mV). NKCC1 was pharmacologically blocked throughout the experiments by bumetanide (10 μM). Action potentials were blocked with TTX (1 μM), and GABA_B_ receptors with CGP 55845 (1 μM). GABA was photolyzed from the carboxy-2-nitrobenzyl (CNB)-caged GABA compound (Invitrogen, Carlsbad, CA, USA). Caged GABA (2.5 mM) was dissolved in the physiological solution and delivered at a flow rate of 1 μl/min to the vicinity of the recorded cell using an UltraMicroPump II syringe pump (WPI, Sarasota, FL, USA) and a syringe with an inner tip diameter of 100 μm. For local photolysis of caged GABA, the 375 nm output of a continuous emission diode laser (Excelsior 375, Spectra-Physics) was delivered to the slice through an Olympus LUMPlanFl 40× water-immersion objective. The UV beam yielded an uncaging spot of ~10 μm diameter that was focused either at the soma or at the dendrite. At each location, the current-voltage relationship was determined by varying the holding potential from −80 to −40 mV (10 mV steps). Control experiments showed that changing the voltage-step protocol from hyperpolarizing–depolarizing to depolarizing–hyperpolarizing did not affect the estimated E_GABA_. Peak current amplitudes were measured and plotted against the holding potential to obtain an estimate of E_GABA_. For each cell, the somatic and dendritic sites were tested in a randomized manner. The gradient (ΔE_GABA_) was defined as the difference of the dendritic value of E_GABA_ and the E_GABA_ at the soma.

### Analysis of Excitatory Miniature Postsynaptic Currents and Biocytin Loading

mEPSCs were recorded from CA3 pyramidal neurons in whole-cell voltage-clamp configuration (−60 mV). Slices were placed in a submerged recording chamber and perfused with the extracellular solution supplemented with bicuculline (10 μM) and TTX (1 μM). Patch pipettes were fabricated from borosilicate glass (Harward Apparatus, UK), and their resistance ranged from 6 to 8 MΩ. The pipette solution consisted of (in mM) CsMeSO_4_, 130; HEPES, 10; EGTA, 0.5; Mg-ATP, 4; Na-GTP, 0.3; QX-314, 5; NaCl, 8; and biocytin, 10; 285 mOsm, pH 7.2. After at least 15 min or stable recording, the electrode was slowly removed, and the slices were fixed in 4% PFA in PBS. Only cells with access resistance that did not exceed 20 MΩ were accepted for analysis. The average frequency and amplitude of these events were analyzed using the MiniAnalysis Program (Synaptosoft).

### Antibodies

Rabbit anti-KCC2 pan polyclonal antibodies (Ludwig et al., 2003) were produced and purified by Innovagen AB and used at a dilution of 1:1,000 for IHC. Secondary antibodies, donkey anti-rabbit conjugated to Alexa Fluor^®^ 568, and goat anti-mouse conjugated to Alexa Fluor^®^ 488 were used at dilutions of 1:400. For visualization of biocytin labeling, streptavidin conjugated to Alexa Fluor^®^ 568 (Thermo Fisher Scientific) was used at dilutions of 1:1,000.

### Immunohistochemistry

PFA fixed brains were cryoprotected in 30% sucrose in PBS and sectioned into 50 μm thickness of slices using Leica CM 3050S Cryostat. IHC was carried out on free-floating slices. Slices were fixed with 4% PFA for overnight at +4°C, washed with PBS and dehydrated with 30%, 50%, and 80% methanol (MeOH), respectively for 30 min in each concentration. Thereafter they were treated with Dents Fixative (80% MeOH, 20% DMSO) for 1 h and washed twice in TBSTD (TBS +0.1% Tween + 5% DMSO). Blocking was carried out with 5% donkey serum and 1% Goat serum in TBSTD ON at +4°C. Primary antibodies were applied in the blocking solution for 48 h at +4°C. Slices were washed three times and secondary antibodies were diluted 1:400 in TBSTD and applied ON (overnight at RT). Slices were then washed with TBSTD twice and mounted onto microscopy glass slides using Prolong Gold mounting medium with DAPI.

### Image Acquisition and Analysis of KCC2 Membrane Expression

Images for all data analyses were obtained with a Zeiss LSM 710 confocal microscope and EC Plan NEOFLUAR 20× and 63× objectives using the Zen Imaging software. Laser lines used were the argon, diode, and helium-neon for imaging fluorescent Alexa-488, Alexa-568, and DAPI/Hoechst, respectively. Stacks of 4–5 images were captured, with a single slice height of 2.09 μm, a resolution of 1,704 × 1,704 (pinhole 1 AU), and a 4-line average. Laser powers, image gain, and other acquisition settings were kept consistent/as same or constant for all images within an experiment. All images were coded and further analyzed blindly. For quantification of plasma membrane-like expression of KCC2, the immunolike intensity at the soma (2–3 μm from cell center) was subtracted from value from 6 to 7 μm (defined by the EGFP expression profile). The resulting values were then normalized with the corresponding values obtained in naïve neurons.

### Cell Culture

Standard dissociated hippocampal culture was prepared from embryonic day (E18) rat (dissociated by enzymatic treatment-0.25% trypsin for 15 min at 37°C) and cultured up to 15 days *in vitro*. The cells were plated on 13 mm polyethyleneimine (PEI, 20 μg/ml) coated coverslips (2 × 10^5^ cells/cm^2^ in Neurobasal medium containing 2% B27 supplement and 0.5 mM L-glutamine (Gibco/Life Technologies). Cultures were fed once a week by changing half of the medium. On DIV3, the cultured hippocampal neurons were transduced with either control-DsRED/EGFP or shRNAGluK2-DsRED/EGFP (see viral vector description above).

### Fluorescence Recovery After Photobleaching (FRAP)

Fluorescence recovery of the GFP–β-actin intensity after photobleaching was performed with a confocal microscope (LSM 710 inverted confocal; Zeiss) at 37°C and with 5% CO_2_ in HBSS (Hank’s buffered saline solution). The cultured hippocampal neurons were infected with either control-DsRED or shRNA_GluK2_-DsRED on DIV3. On DIV13–14, eGFP–β-actin plasmid was transfected using CombiMag Magnetofection Transfection Reagent (OZBIOSCIENCES). In each group, DsRED positive cells were chosen to bleach eGFP–β-actin in dendritic spines. Time-lapse images were taken with a 63×/0.90 NA dipping water objective and 5× digital zoom. The microscope was controlled by ZEN software, and the settings were as follows: format 256 × 256, speed 12, unidirectional with one line averaging, and pinhole of 200 with 1.0 airy units (AU). The frame of 26.9 × 26.9 μm, including the region of interest (the whole spine), was imaged five times (pre bleach baseline) followed by photobleaching that was achieved with one scan (with total 100 iterations, total laser power = ~10 mW) of the region of interest. Imaging of the area was resumed immediately after the photobleaching in a total of 300 cycles with 1.0-s interval. The intensity of the bleached area was normalized to a neighboring non-bleached dendritic area to diminish error caused by photobleaching during the monitoring period. The pre bleach value was normalized to 1.0.

### Western Blot

On DIV15, hippocampal cultures were rinsed in ice-cold PBS and homogenized in ice-cold radioimmunoprecipitation (RIPA) lysis buffer (150 mM NaCl, 1% Triton X-100, 0.5% deoxycholate, 0.1% SDS, and 50 mM Tris-HCl, pH 8.0) with protease inhibitor cocktail (cOmplete; Roche) and phosphatase inhibitor cocktail (PhosSTOP; Roche), centrifuged at 15,000× *g* for 15 min at 4°C. Protein concentrations were determined using the DC Protein Assay kit (Bio-Rad Laboratories Inc., Hercules, CA, USA). Samples were eluted in SDS/2-mercaptoethanol sample buffer and denatured for 15 min at room temperature, loaded on SDS-PAGE for separation then transferred to polyvinylidene difluoride (PVDF) membrane. Blots were probed with the chicken anti-KCC2b (Markkanen et al., 2014), mouse anti-cofilin antibody (1:1,000 in TBST + 2.5% BSA, #sc-376476 Santa Cruz Biotechnology, Dallas, TX, USA), rabbit anti-phosphocofilin antibody (Serine-3; 1:1,000 in TBST + 2.5% BSA #sc21867-R, Santa Cruz Biotechnology, Dallas, TX, USA), rabbit anti-GluK2/3 (anti-GluR6/7) antibody (1:2,000 in TBST + 2.5% BSA #04-921, Millipore clone NL9), rabbit anti-4.1N antibody (1:1,000 in TBST + 2.5% BSA from Dr. Kari Keinänen) mouse anti-α tubulin (1:5,000 in TBST + 2.5% BSA, #T9026, Sigma) primary antibodies and HRP-conjugated donkey anti-rabbit and donkey anti-mouse (GE Healthcare) or goat anti-chicken IgY (Abcam) secondary antibodies, followed by developing with ECL Plus (GE Healthcare) and visualized with LAS-3000 (Fujifilm). Band densities were analyzed with ImageJ software.

### Statistical Analysis

Prism 8 (Graph Pad) was used for all graphs and statistical analyses. Data are presented as mean ± SEM, and quantitative comparisons were based on Student’s *t*-test, or Kolmogorov–Smirnov test as well as one way ANOVA using Tukey’s multiple comparison test. *P* < 0.05 was considered significant and indicated in the figures.

## Results

### GluK2 Silencing in Hippocampal CA3 Pyramidal Neurons Downregulates KCC2 Expression and Chloride Extrusion Efficiency *in vivo*

**Figure 1:**
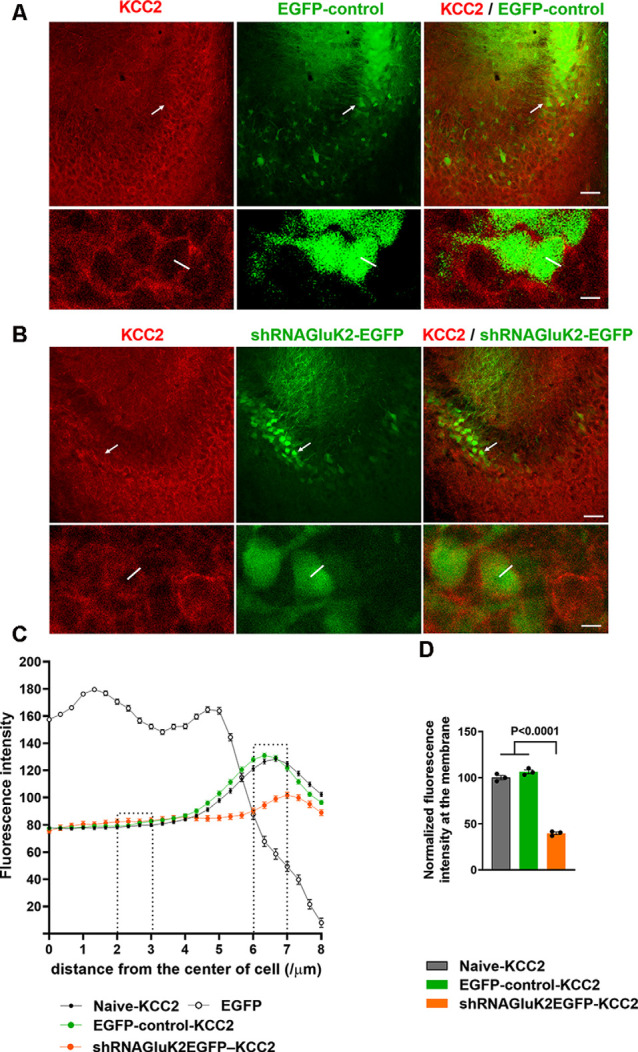
Downregulation of K^+^-Cl^−^ cotransporter (KCC2) after acute silencing of GluK2 in CA3 hippocampal neurons *in vivo*. **(A,B)** KCC2 immunostaining (in red) of CA3 hippocampal section from animals transduced with EGFP-control **(A)** or shRNA_GluK2_-EGFP **(B)** lentivirus. Lower panel shows a high magnification single confocal section of images of cells marked with a white arrow in the upper panel. **(C)** Graph showing the line intensity profile from the center of the cell. Examples of lines are shown in the inserts in **(A)**. **(D)** Bar graph showing the normalized cumulative fluorescence intensity. The regions between 2–3 μm and 6–7 μm from the center of the cell were used for quantification. Data are presented as mean ± SEM (*p* < 0.0001 considered significant). Scale bar: 100 μm (low magnification panels) and 20 μm (insert).

Previous results have shown that GluK2 expression is important for physiological surface expression of KCC2 and corresponding chloride transport in cortical neurons (Mahadevan et al., 2014; Pressey et al., 2017; Garand et al., 2019). To validate the role of GluK2 in regulating KCC2 *in vivo*, we used lentiviral shRNA (shRNA_GluK2_-EGFP) for local silencing of GluK2 in WT mice CA3 pyramidal neurons, where endogenous GluK2 is strongly expressed in dendrites (Fièvre et al., 2016). Control animals were injected with lentivirus encoding for EGFP. Stereotaxic injections of viruses were performed on P10–12 animals followed by immunohistochemistry and *ex vivo* recordings at P19–20. The subcellular distribution of KCC2 in CA3 pyramidal neurons was visualized using KCC2 antibodies. First, the specificity of staining was verified using knockout tissue (Supplementary Figure S1). KCC2 immunolabeling showed a strong perisomatic-like staining pattern in naïve neurons (100 ± 2.3% *n* = 400 cells) as well as in the neurons infected by control EGFP virus (106.23 ± 2.1% *n* = 200 cells, three animals; Figures 1A,C,D). In contrast, neurons infected with shRNA_GluK2_-EGFP had a significantly reduced perisomatic-like immunoreactivity pattern (39.75 ± 1.37% *n* = 200 cells, three animals; *p* < 0.001; Figures 1B,C,D).

**Figure 2:**
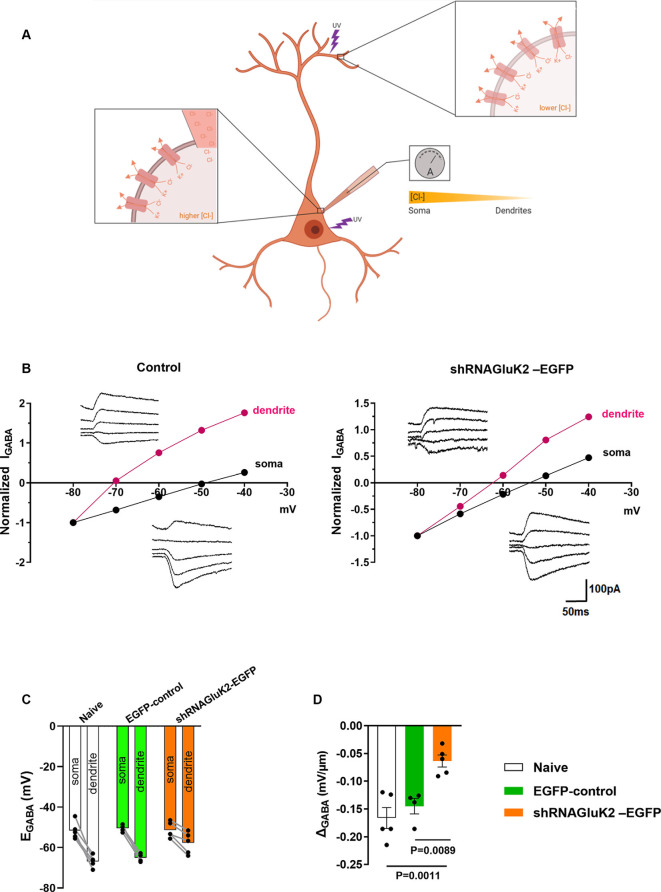
GluK2 silencing reduces chloride extrusion efficacy in CA3 hippocampal dendrites. **(A)** Schematic representation of somatodendritic intracellular Cl^−^ gradient quantification using local photolysis of caged GABA. **(B)** Example traces of GABA_A_responses (inserts) from soma and dendrites shown with their corresponding I-V plots. For control (EGFP) expressing only neurons (left) and shRNA_GluK2_ expressing neurons (right). **(C)** Graph showing the E_GABA_ values in dendrite and soma for Naïve, EGFP-control, and shRNA_GluK2_-EGFP transduced neurons. **(D)** Somato-dendritic gradient quantification presented as mean ± SEM of naïve, EGFP-control and shRNA_GluK2_, respectively (*p* < 0.05 considered significant).

To test the effect shRNA_GluK2_-EGFP on chloride extrusion in CA3 pyramidal neurons, we measured the somatodendritic gradient of E_GABA_ using whole-cell patch-clamp and local uncaging of GABA at both soma and distal dendrite (Khirug et al., 2005; Figure 2A). This gradient was significantly reduced in GluK2 silenced neurons (ΔE_GABA_ = −0.0637 ± 0.01086 mV/μm; *n* = 5 neurons, three animals) compared to EGFP-controls (ΔE_GABA_ = −0.145 ± 0.01389 mV/μm; *n* = 4 neurons, three animals, *p* = 0.0022) and to naïve neurons (ΔE_GABA_ = −0.1662 ± 0.01882 mV/μm; *n* = 5 neurons, four animals, *p* = 0.0015; Figures 2B–D). Thus, KCC2 efficacy was compromised by the acute GluK2 silencing. This is reflected by the inefficient extrusion of intracellular Cl^−^ disclosed by somatic Cl^−^ loading in CA3 pyramidal neurons. Together, these findings show that acute silencing of GluK2 leads to a decrease in the membrane expression and function of KCC2 in CA3 pyramidal neurons.

### Local GluK2 Silencing Impairs the Development of Dendritic Spines and Excitatory Transmission in CA3 Pyramidal Neurons *in vivo*

**Figure 3:**
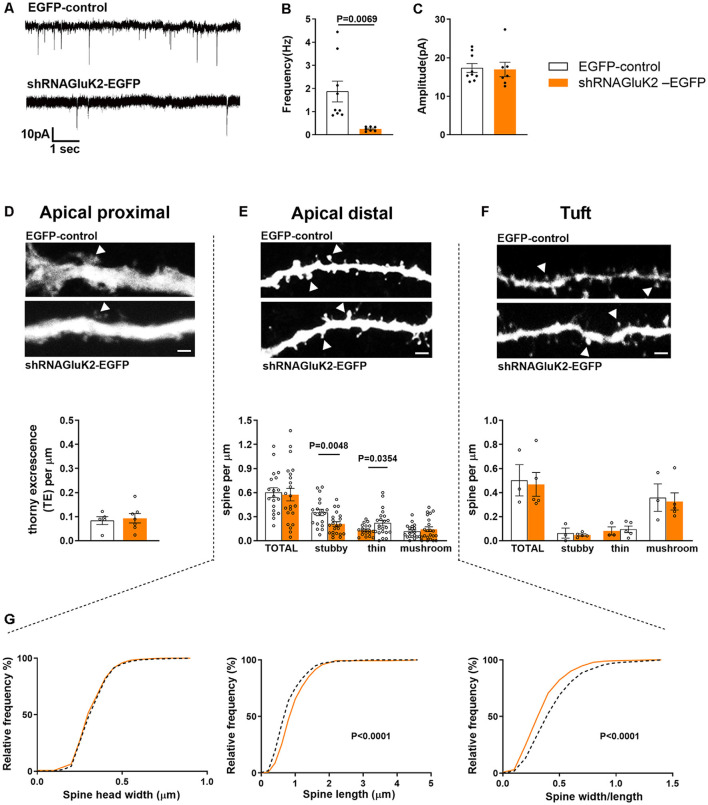
*In vivo* GluK2 silencing results in a low frequency of mEPSCs and aberrant spine morphology in CA3 dendritic spines. **(A–C)** Reduced number of functional synapses in dendrites of shRNA_GluK2_ expressing neurons. **(A)** Example traces of mEPSCs from EGFP-control and shRNA_GluK2_ expressing CA3 pyramidal neurons. Pooled data for the frequency **(B)** and amplitude **(C)**. Data are presented as mean ± SEM (*p* < 0.001 considered significant). (**D–F** upper panel) Example dendritic segments from apical proximal, distal, and tuft regions used for dendritic spine morphology analysis. The upper panel shows GFP-control and lower panel shRNA_GluK2_ respectively. Calibration bar: 5 μm. (**D–F** lower panel) Quantification of spine density in apical proximal (thorny excrescence; **D**), distal **(E)**, and Tuft **(F)** dendritic segments. Panel **(G)** shows the frequency distribution of the dendritic spine head width, spine length, and width/length ratio from apical distal dendritic segments. White bar and segmented lines represent data from EGFP-control cells. Orange-colored bars and filled lines represent data from shRNA_GluK2_ expressing neurons.

To investigate the role of endogenous GluK2 in the maturation and maintenance of excitatory transmission and dendritic spines, we performed parallel functional and morphometric analysis of hippocampal CA3 pyramidal neurons lacking GluK2. Control (EGFP) and shRNA_GluK2_-EGFP lentiviruses were injected in the CA3 region of the hippocampus in WT mice at P10–12. Whole-cell patch-clamp recordings (P19–20) were used to record mEPSCs of EGFP-labeled CA3 pyramidal neurons in the presence of TTX (1 μM) and Bicuculline (10 μM), to block voltage-gated sodium channels and GABA-A receptors, respectively. During the recordings, the neurons were filled with biocytin for *post hoc* morphological analysis. Neurons infected with shRNA_GluK2_-EGFP showed a significantly lower frequency of mEPSCs compared to EGFP-controls (shRNA_GluK2_-EGFP 0.25 ± 0.04 Hz; *n* = 7 neurons, four animals; EGFP 1.87 ± 0.45 Hz; *n* = 9 neurons, five animals; *p* = 0.007; Figures 3A,B). No difference in the amplitudes of mEPSCs were detected between the groups (shRNA_GluK2_-EGFP 16.99 ± 1.87 pA; *n* = 7 neurons, four animals; EGFP 17.36 ± 1.17 pA; *n* = 9 neurons, five animals; *p* = 0.866; Figures 3A,C).

*Post hoc* 3D morphometric analysis was performed by assessing the dendritic protrusion along the apical proximal oblique, distal oblique, and tuft dendrites (Figure 3D). We assessed the thorny excrescences and other dendritic protrusions/spines based on standard parameters in three categories: mushroom, stubby and thin-type spines (Rodriguez et al., 2008). This analysis revealed no difference in the total density of thorny excrescences or other dendritic spines between EGFP-control and GluK2 deficient dendrites (Figures 3D–F). However, there was a change in the spine morphology: in apical distal dendrites, the density of stubby spines was significantly lower in shRNA_GluK2_-EGFP expressing neurons as compared to EGFP controls (EGFP-control: 0.35 ± 0.05 vs. shRNA_GluK2_-EGFP: 0.21 ± 0.05) while the density of thin spines was higher; (EGFP-control: 0.13 ± 0.04 vs. shRNA_GluK2_ EGFP: 0.22 ± 0.04; EGFP-control, *n* = 5 neurons, 19 dendritic segments vs. shRNA_GluK2_-EGFP, *n* = 8 neurons, 22 dendritic segments; Figure 3E). In addition to this, the length of spines and the length to head ratio were significantly higher in GluK2-deficient distal dendrites as compared to EGFP-control (Length: EGFP-Control: 0.87 ± 0.02 μm vs. shRNA_GluK2_-EGFP: 1.0 ± 0.02 μm; *p* < 0.0001; width/length; EGFP-Control: 0.48 ± 0.01 vs. shRNA_GluK2_-EGFP: 0.39 ± 0.01; Figure 3G).

Interestingly, the spine phenotype in GluK2 deficient neurons is reminiscent of that reported previously for KCC2 hypomorphic mice (Tornberg et al., 2005). To directly compare the effects of local silencing of KCC2 and GluK2 on dendritic spines, we infected KCC2^ flox/flox^ mice with AAV8-CaMKIIa-mCherry-Cre virus to delete KCC2 expression in CA3 pyramidal neurons at the corresponding developmental time point (P10–12). One week after viral transduction, the mice were processed for morphological analysis.

Similar to GluK2, KCC2 deficiency had no effect on the total density of spines (EGFP-control: 0.60 ± 0.07 μm; *n* = 5 neurons, 19 dendritic segments vs. mCherry-Cre: 0.69 ± 0.08 μm; *n* = 4 neurons, 16 dendritic segments; *p* = 0.33), however the density of thin spines was significantly higher as compared to controls (EGFP-control: 0.13 ± 0.016 μm vs. mCherry-Cre: 0.27 ± 0.04 μm; *p* = 0, 01; Supplementary Figures S2A,B). Further, the head diameter of dendritic spines was not different between groups (Supplementary Figure S2C; EGFP-control: 0.34 ± 0.04 μm vs. mCherry-Cre 0.35 ± 0.01 μm; *p* = 0, 56), but the length of spines was significantly higher in KCC2 deficient neurons as compared to control (EGFP-control: 0.87 ± 0.02 μm vs. mCherry-Cre: 1.0 ± 0.03 μm; *p* < 0.0001; Supplementary Figures S2A,B). Together, these data demonstrate that GluK2 silenced and KCC2-depleted CA3 pyramidal neurons have aberrant dendritic spines with similar morphological phenotype.

### GluK2 Expression Regulates Dendritic Cofilin and 4.1N as Well as the Stability of F-Actin in Dendritic Spines

We have previously shown that KCC2 interacts with intracellular proteins such as 4.1N and cofilin to regulate the formation of glutamatergic synapses and dendritic spines (Li et al., 2007; Llano et al., 2015). Furthermore, previous results have shown that 4.1N expression is strongly reduced in GluK2^−/−^ mice (Copits and Swanson, 2013). To assess whether the expression of 4.1N and cofilin are altered in response to local inactivation of GluK2 expression, we conducted Western blot analysis using cultured hippocampal neurons.

**Figure 4:**
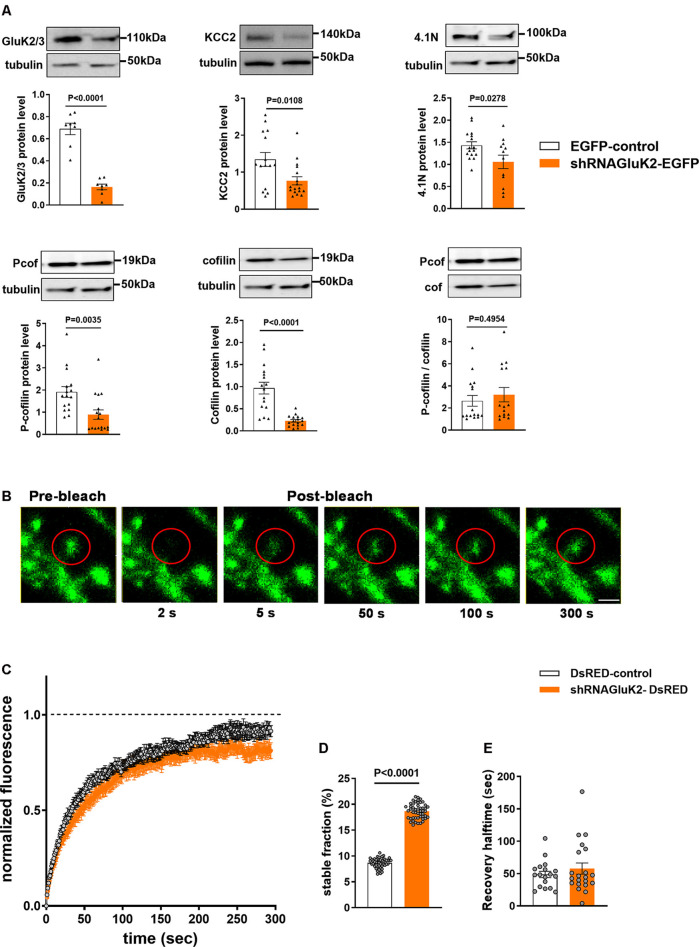
Effect of GluK2 on the expression of KCC2 interacting proteins 4.1N and cofilin as well as β-actin dynamics. **(A)** The upper panel shows representative Western blot analysis from hippocampal cultured neurons transduced with either EGFP-control or shRNA_GluK2_ virus using antibodies against GluK2/3, KCC2, 4.1N as well as pan- and phospho-specific forms of cofilin. The corresponding bar graphs representing the normalized expression of GluK2/3, KCC2, 4.1N, cofilin and Pcof/cof ratio from EGFP-control and GluK2 silenced cultured hippocampal neurons, respectively. Data are presented as mean ± SEM (<0.05, *p* < 0.001 considered significant). **(B)** Time-lapse images of fluorescence recovery after photobleaching (FRAP) in a control dendritic spine expressing β-actin-EGFP (Scale bar: 2 μm). **(C)** Averaged FRAP curves depicting the β-actin turnover rate from control and GluK2-silenced hippocampal neurons. **(D)** Histogram of a stable fraction of β-actin-EGFP and **(E)** recovery half time from control and GluK2-silenced hippocampal neurons (18 spines for control and 21 spines for shRNA_GluK2_). Data are presented as mean ± SEM (*p* < 0. 0001 considered significant).

First, we assessed the efficacy of shRNA_GluK2_-EGFP to silence GluK2 expression in primary hippocampal culture using antibodies against GluK2/3. As expected, shRNA_GluK2_-EGFP lentiviral transduction significantly reduced the expression of GluK2/3 in cultured hippocampal neurons (Figure 4A, 76.24 ± 5.8% of EGFP-control; *n* = 8 samples from three independent experiments; *p* < 0.0001). Similarly, shRNA_GluK2_-EGFP treatment significantly decreased the expression of KCC2 in cultured hippocampal neurons (Figure 4A, 42.86 ± 21.2% of EGFP-control; *n* = 17 samples from three independent experiments; *p* = 0.01).

Western blot results showed a significant reduction in both 4.1N, cofilin and phospho-cofilin expression after GluK2 silencing in hippocampal neurons (Figure 4A; 4.1N 26.1 ± 16% of EGFP-control, *n* = 12 samples from three independent experiments; *p* = 0.03; cofilin, 23.46 ± 13.2% EGFP control, *n* = 16 samples from three independent experiments *p* < 0.0001, phospho-cofilin; 46.6 ± 3.2% of EGFP control *n* = 16 samples from three independent experiments, *p* = 0.003) Interestingly, we did not find significant changes in the relative cofilin phosphorylation (Figure 4A; Pcof/cof ratio, 17.3 ± 80% of EGFP-control, *n* = 16; *p* = 0.50). All these effects are consistent with a change in actin regulatory network in GluK2 deficient neurons.

To directly test whether acute loss of GluK2 affects actin dynamics in dendritic spines we performed fluorescence recovery after photobleach (FRAP) analysis of β-actin-EGFP in GluK2 deficient vs. control neurons (Star et al., 2002; Koskinen et al., 2012; Llano et al., 2015; Figure 4B). We found that in dendritic spines of cultured neurons infected by control virus, expressing only DsRED, the recovery of the fluorescence intensity after bleaching was close to 100% within a minute (Figure 4C). However, in dendritic spines of GluK2 silenced neurons (expressing shRNA_GluK2_-DsRED) β-actin-EGFP fluorescence recovered to ~80% of the initial level, indicating a higher ratio of a stable pool of actin in GluK2 deficient dendritic spines (Figure 4D; Control stable fraction: 0.0865 ± 0.001315 *n* = 18 spines; shRNA GluK2: 0.1865 ± 0.002081 *n* = 21 spines; *p* < 0.0001). In contrast, the silencing of GluK2 did not affect the half-time of fluorescence recovery (Figure 4E; Control t1/2: 48.59 ± 4.922 *n* = 18 spines and shRNA_GluK2_ t1/2: 57.7 ± 8.632 *n* = 21 spines; *p* = 0.3862).

### KCC2 Overexpression Rescues Dendritic Spine Reduction Induced by GluK2 Silencing

**Figure 5:**
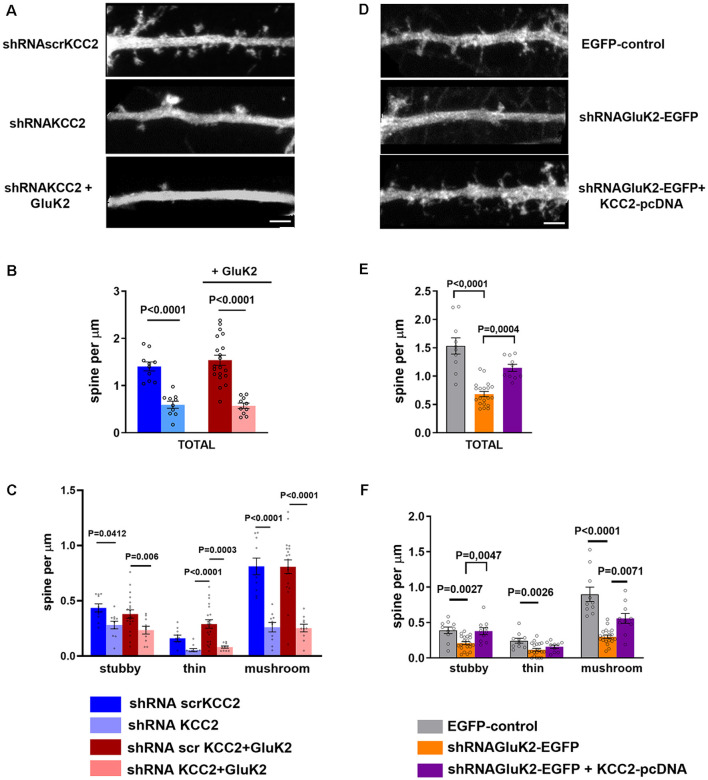
KCC2 overexpression rescues GluK2 depletion induced aberrant spine morphology. **(A)** High magnification photomicrograph of dendritic segments expressing scrambled shRNA_scrKCC2_, shRNA_KCC2_ and shRNA_KCC2_ + GluK2 (scale bar: 5 μm). **(B)** Quantification of dendritic spine density showing a significant reduction in KCC2-depleted cultured hippocampal neurons. Overexpression of GluK2 however was not able to rescue this reduction. **(C)** Pooled data on the effect of KCC2 knockdown in different types of spines. Data are presented as mean ± SEM (*p* < 0.05, *p* < 0.001considered as significant). **(D)** High magnification photomicrograph of dendritic morphology in neurons expressing EGFP-control and shRNA_GluK2_-EGFP and well as shRNA_GluK2_ + KCC2 pcDNA (scale bar: 5 μm). **(E)** Quantified data illustrating that GluK2 silenced hippocampal neurons have a significantly lower number of dendritic spines in total in comparison with EGFP-control and this reduction is rescued by the overexpression of KCC2. **(F)** GluK2 silenced hippocampal neurons have a significantly lower number of dendritic spines of all subclasses in comparison with control. Overexpression of KCC2 can rescue the effect of shRNA_GluK2_ on dendritic spine morphology. Data are presented as mean ± SEM (*p* < 0.05, *p* < 0.001 considered significant).

In order to confirm the significance of GluK2-KCC2 interaction for dendritic spine formation/maturation, we performed rescue experiments in primary hippocampal neurons. In contrast to the *in vivo* data, shRNA-mediated knockdown of KCC2 resulted in a significant reduction in the total density of dendritic spines in cultured neurons (control: 1.37 ± 0.15 μm; Supplementary Figure S3; shRNA_scr_: 1.41 ± 0.09 μm; shRNA _KCC2_: 0.59 ± 0.07 μm; *n* = 10/group; *p* < 0.001; Figures 5A,B). Among different type of dendritic spines, mushroom type spines were the most affected with a significant reduction as compared to control and shRNA_scr_ (shRNA_scr_: 0.87 ± 0.13 μm; *n* = 10; shRNA_KCC2_: 0.26 ± 0.04 per μm 10 dendritic segments; *p* < 0.0001; Figure 5C). Lentiviral overexpression of GluK2 had no effect on the spine density in control neurons (1.54 ± 0.11 μm, *n* = 10; Supplementary Figure S3) and was not able to rescue the reduced density of dendritic spines after KCC2 knock down (shRNA_KCC2_ + GluK2 0.57 ± 0.06 μm, *n* = 10; Figures 5A,B).

Interestingly, we found that knockdown of GluK2 (shRNA_GluK2_-EGFP) was associated with a similar reduction of the total number of dendritic spines density in cultured hippocampal neurons as compared to KCC2 knockdown (control: 1.53 ± 0.14 μm vs. shRNA_GluK2_, 0.68 ± 0.05 μm, *n* = 10 for both groups; *p* < 0.0001; Figures 5D,E). Similarly to the effect of KCC2 downregulation this effect was mainly due to reduction of mushroom type spines from 0.89 ± 0.10 μm (10 dendritic segments) to 0.29 ± 0.02 μm (10 dendritic segments; *P* < 0.001; Figure 5F). Most strikingly in neurons that expressed both the shRNA_GluK2_ and KCC2 expression plasmid, the density of dendritic spines had recovered close to control levels (1.14 ± 0.06 μm, *n* = 10; *p* = 0.0004 compared to shRNA_GluK2_). This effect was seen over all type of spines (Figures 5E,F).

Our results demonstrate that the interaction between KCC2 and GluK2 is an essential component for normal dendritic spine formation.

## Discussion

Evidence from GluK2^−/−^ and KCC2 hypomorphic mice point to a significant role of both GluK2 and KCC2 in the normal development of glutamatergic circuitry in the brain (Marchal and Mulle, 2004; Tornberg et al., 2005; Lanore et al., 2012; Li et al., 2013). However, the exact mechanisms underlying these developmental effects are not fully understood. In this study, we show that GluK2 deficiency results in parallel loss of KCC2 surface expression and aberrant spine morphology *in vivo*, which can be fully rescued *via* KCC2 overexpression. These data suggest that the interaction of KCC2 with GluK2 is critical in the maturation and maintenance of dendritic spines.

Previous results have shown that GluK2 both binds and regulates the surface expression of the KCC2 (Mahadevan et al., 2014), which is important for the maintenance of chloride homeostasis in hippocampal neurons (Mahadevan et al., 2014). In agreement with the *in vitro* results, we found that the downregulation of GluK2 in CA3 pyramidal neurons *in vivo* caused a redistribution of KCC2 from the plasmalemma as well as a decreased chloride extrusion efficacy. These results confirm that GluK2 plays an important role in regulating KCC2 function in CA3 pyramidal neurons *in vivo*.

Transient activation of GluK2 containing KAR has been shown to induce dendritic spine enlargement and potentiation of glutamatergic synapses (Petrovic et al., 2017). On the other hand, the absence of GluK2 is associated with a significant delay in the development of mossy fiber input to CA3 neurons (Marchal and Mulle, 2004; Lanore et al., 2012). These previous results and the stabilizing effect of GluK2 on KCC2 distribution and expression prompted us to investigate the role of GluK2 on the dendritic spine morphology of the CA3 principal neurons. Downregulation of GluK2 had a strong impact on the morphology of dendritic spines in both primary hippocampal cultures *in vitro* and CA3 pyramidal neurons *in vivo*. Interestingly, significant downregulation of total spine density was only observed *in vitro*, probably reflecting the rapid turnover of the spines under culture conditions. The milder effect on dendritic spines observed *in vivo* compared to *in vitro* could be also derived from the more variable efficacy of infection *in vivo*. Nevertheless, the effects observed in GluK2 knockdown neurons were significant implying an important role for GluK2 dependent signaling in dendritic spines maturation. This is also similar to the effect of KCC2 ablation *in vivo* shown previously by us and other groups (Tornberg et al., 2005; Li et al., 2007). Strikingly, the effect of GluK2 downregulation in spine morphology could be rescued by overexpression of KCC2. In contrast, the abnormal spine morphology induced by the decreased expression of KCC2 was not rescued by overexpression of GluK2. These results suggest that the spine morphological changes are attributed to the GluK2-dependent decrease of KCC2 expression at the plasma membrane.

Although, the metabotropic signaling of KAR has been implicated in structural plasticity of dendritic spines (Petrovic et al., 2017) the intracellular mechanism for this effect is not known. Recent work has shown that both ionotropic and metabotropic signaling elicited by KAR activation can regulate KCC2 function (Garand et al., 2019). This could be mediated by an increase in the stability and/or trafficking of KCC2 at the plasma membrane and is in line with the effects reported in this work and previous publications. The data presented here makes it plausible that the mechanism is previously shown to be involved in the effect of KCC2 in spine morphology (Li et al., 2007; Kaila et al., 2014; Llano et al., 2015) would be also downstream of kainate receptors. The involvement of KAR metabotropic signaling in this mechanism is an exciting possibility that should be investigated soon.

The stability of glutamatergic synapses and dendritic spines is dependent on a delicate balance within a network of proteins. The morphogenic role of KCC2 in dendritic spine maturation is attributed to the interaction with proteins β-PIX and 4.1N (Li et al., 2007; Llano et al., 2015), which may link KCC2 expression and distribution with the regulation of dynamics of the actin cytoskeleton and glutamate receptors. In KCC2-deficient neurons, the stability of F-actin is dramatically increased (Chevy et al., 2015; Llano et al., 2015) and trafficking of AMPA receptors to the synaptic membrane is impaired (Gauvain et al., 2011; Fiumelli et al., 2013; Chevy et al., 2015). Both effects involve changes in the functional state of the actin filament severing protein cofilin (Hotulainen et al., 2009; Hotulainen and Hoogenraad, 2010; Borovac et al., 2018), which is crucial for regulating the stability of the actin cytoskeleton as well as the stability and formation of dendritic spines (Yoshihara et al., 2009; Calabrese et al., 2014).

Absence of GluK2 led to changes in the expression of synaptic proteins involved in glutamate receptor trafficking and actin cytoskeleton dynamics that are also downstream of the role of KCC2 in the spine maturation. Although GluK2 silencing had no significant effects on the phosphorylation of cofilin, there was a very strong reduction of total cofilin expression yielding low levels of total phosphor-cofilin in the spines. These results are in line with the abnormal spine morphology found in GluK2-deficient neurons and also suggest that GluK2 expression in spines is important for the proper dynamics of the actin cytoskeleton. Although GluK2 has been previously identified to interact with actin regulatory proteins such as profilin (Mondin et al., 2010) and to regulate actin-dependent morphogenetic processes in neurons (Tashiro et al., 2003; Marques et al., 2013; Sakha et al., 2016; Petrovic et al., 2017; Jack et al., 2019), direct evidence on KAR dependent changes in actin dynamics are lacking. Indeed, when we assessed the effect of GluK2 deficiency on the treadmilling of actin filaments in dendritic spines we found a significant increase in the stable pool of β-actin. This is consistent with a decrease in the active form of cofilin.

The changes in spine morphology were associated with a loss of functional glutamatergic inputs in GluK2 deficient neurons, evidenced by a robust decrease in the frequency of mEPSCs. The KAR contribution to miniature synaptic currents in CA3 pyramidal neurons is low, suggesting that the observed effect predominantly depends on the loss of AMPA-R mediated synaptic inputs. When assessing the expression of GluA1 and 2 we found that decreasing GluK2 expression significantly decreases the expression of GluA2 but not that GluA1. The actin-binding protein 4.1N interacts with both KCC2 and glutamate receptors, and its expression is significantly downregulated in GluK2^−/−^ mice (Copits and Swanson, 2013). Consistently, we detected a significant reduction of 4.1N protein expression when we assessed the total expression of this protein in cultures transduced with GluK2 shRNA. Thus, the interaction of KCC2 with GluK2 may be important for maintaining a correct balance in the dynamics of glutamate receptor trafficking (Chevy et al., 2015) and actin cytoskeleton dynamics through the signaling cascade involving 4.1N.

In conclusion, we propose an important novel role of the interaction of the KAR subunit GluK2 with KCC2 in the formation of glutamatergic synapses and dendritic spines. The results presented here suggest that KCC2 would be downstream of GluK2, coupling KAR mediated glutamatergic signaling to changes spine morphology. A question that remains is whether this mechanism involves parallel or congruent ionotropic and/or metabotropic signaling cascades. Elucidation of the detailed mechanism will not be simple partly due to the complex interplay of chloride dependent and independent interaction with cytoskeleton and glutamate receptor trafficking dynamics.

Abnormal morphology of dendritic spines is a feature associated with psychiatric disorders and developmental disability (Bernardinelli et al., 2014). Loss of function mutations and alterations in RNA editing site of the *Grik2* gene encoding the GluK2 are associated with intellectual disability and autism spectrum disorders (Jamain et al., 2002; Córdoba et al., 2015; Guzmán et al., 2017). Interestingly, increasing the functional expression of KCC2 to treat developmental disorders such as Autism and Rett syndrome has recently gained considerable attention. Currently, there is no precise understanding of the mechanisms behind the positive effects of KCC2 expression. The mechanism proposed in this study suggests that the specific interaction of KCC2 with glutamate receptor subunits could be a therapeutically relevant target.

## Data Availability Statement

The raw data supporting the conclusions of this article will be made available by the authors, without undue reservation.

## Ethics Statement

The animal study was reviewed and approved by Animal Care and Use Committee, University of Helsinki.

## Author Contributions

SKe, SKh, EDi, MS, SS, and EO performed the experiments. SKe, SKh, SL, and CR designed the experiments and wrote the manuscript. SKe and SKh analyzed the data. TT and EDe contributed reagents, materials, and analytical tools.

## Conflict of Interest

The authors declare that the research was conducted in the absence of any commercial or financial relationships that could be construed as a potential conflict of interest.
