# Supplementary material for: The Kainate Receptor Subunit GluK2 Interacts With KCC2 to Promote Maturation of Dendritic Spines
Source: Front Cell Neurosci. 2020 Aug 26;14:252. doi: 10.3389/fncel.2020.00252 (PMC7479265; doi:10.3389/fncel.2020.00252)
Supplement: Supplementary file 1 [file Data_Sheet_1.PDF]

Supplementary Material

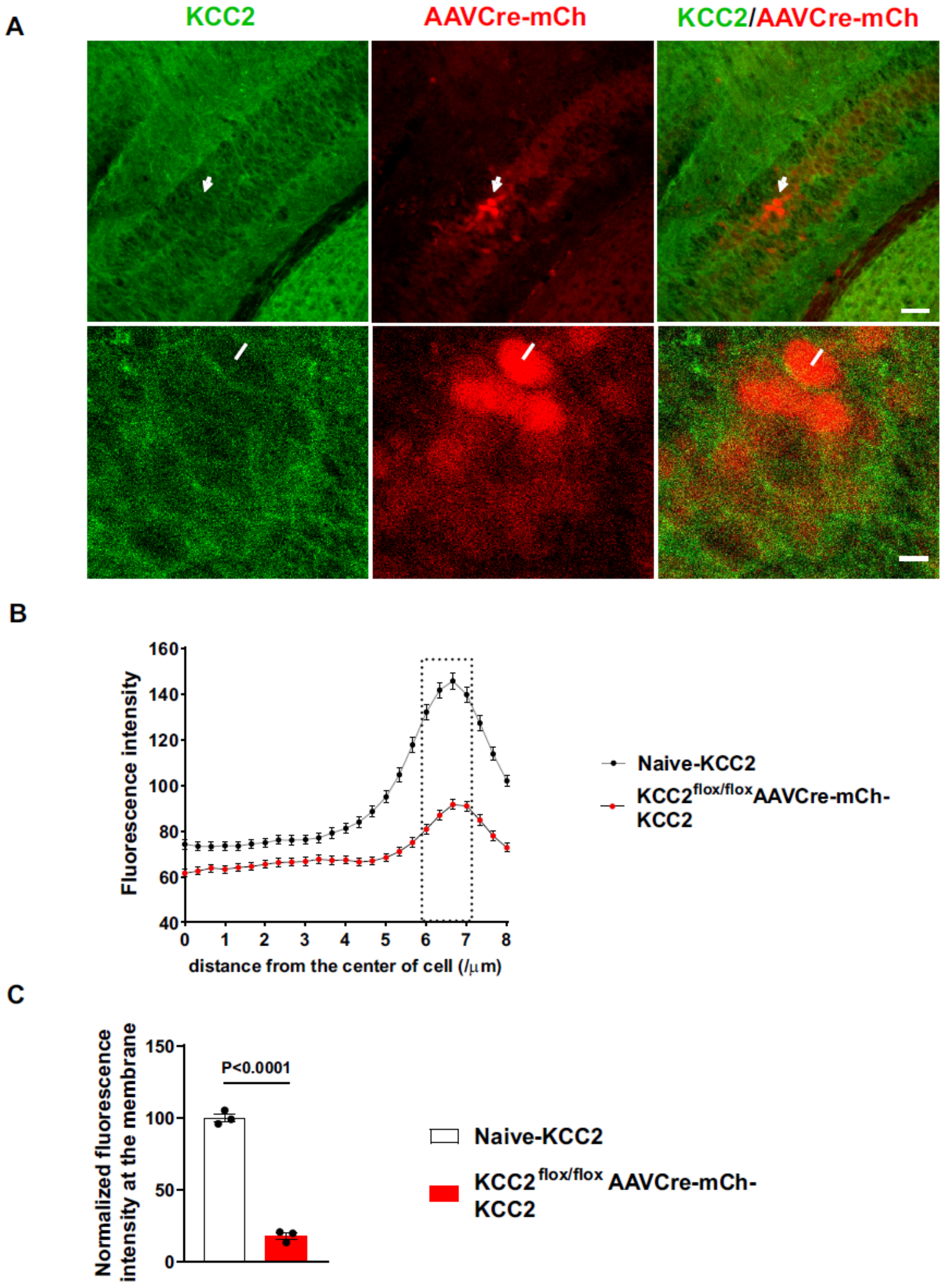

Supplementary Figure 1: Test of specificity of KCC2 staining in CRE expressing KCC2-Floxed CA3 neurons. **(A)** KCC2 immunostaining (in green) of CA3 hippocampal section from animals transduced with AAV8-CaMKIIa.Cre-mCh (AAVCRE-mCh) virus. Lower panel shows high magnification of single section confocal images from cells marked with white arrows in the upper panel. **(B)** Graph showing the line intensity profile from the center of the cell. Example of lines are shown in the lower panel in A. **(C)** Bar graph showing quantification of the normalized cumulative fluorescence intensity between, naïve and KCC2 depleted neurons. Data are presented as mean ± SEM ($p < 0.0001$ was considered significant). Scale bar 100 μm (low magnification panels) and 20 μm (high magnification panels).

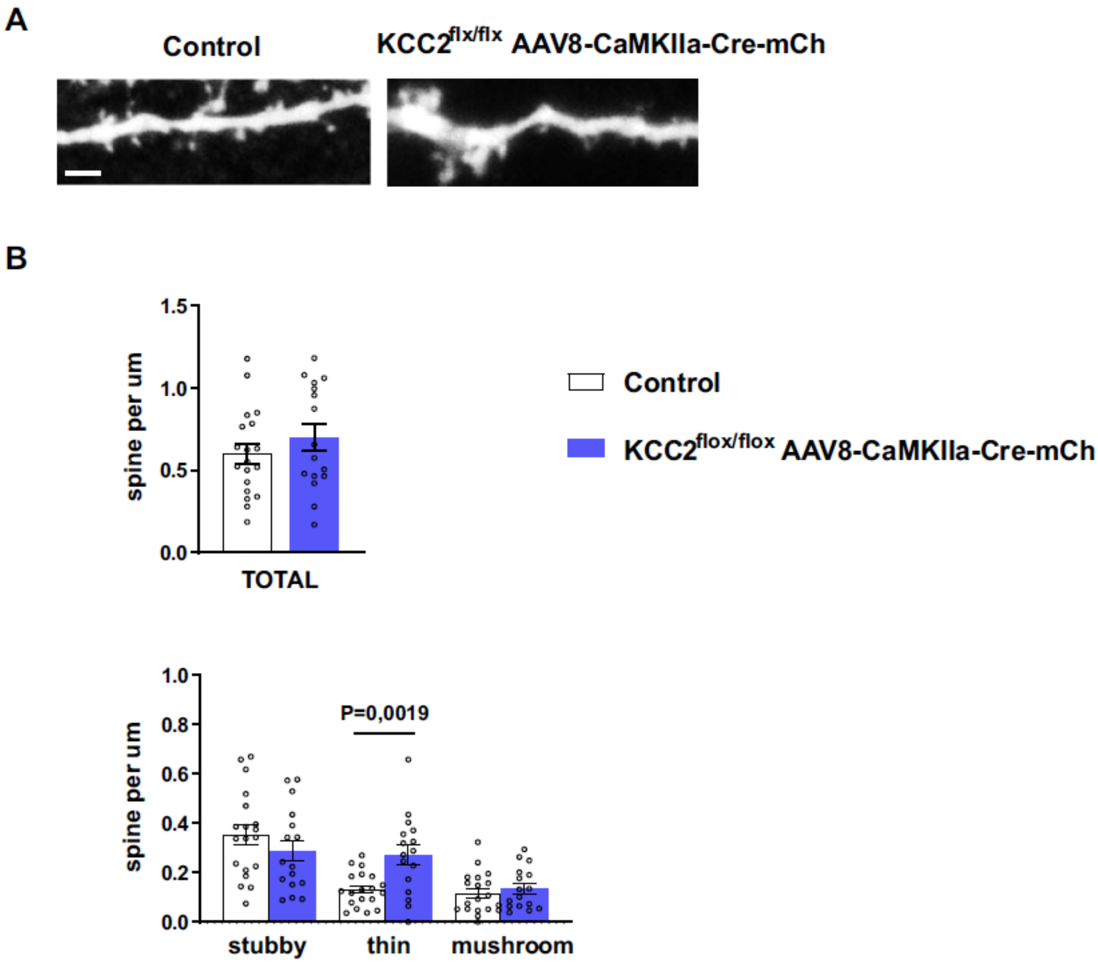

Supplementary Figure 2: KCC2 deletion results in aberrant morphology of CA3 dendritic spines. **(A)** Examples of dendritic segments used for dendritic spine morphology analysis from: KCC2$^{flox/flox}$ transduced with either control or AAV8-CaMKIIa-mCherry-Cre virus. Scale bars: 5 µm. **(B)** Quantification of dendritic spine density calculated as spines/µm of dendrite. Data are represented as mean ± SEM ($p<0.05$ considered significant)

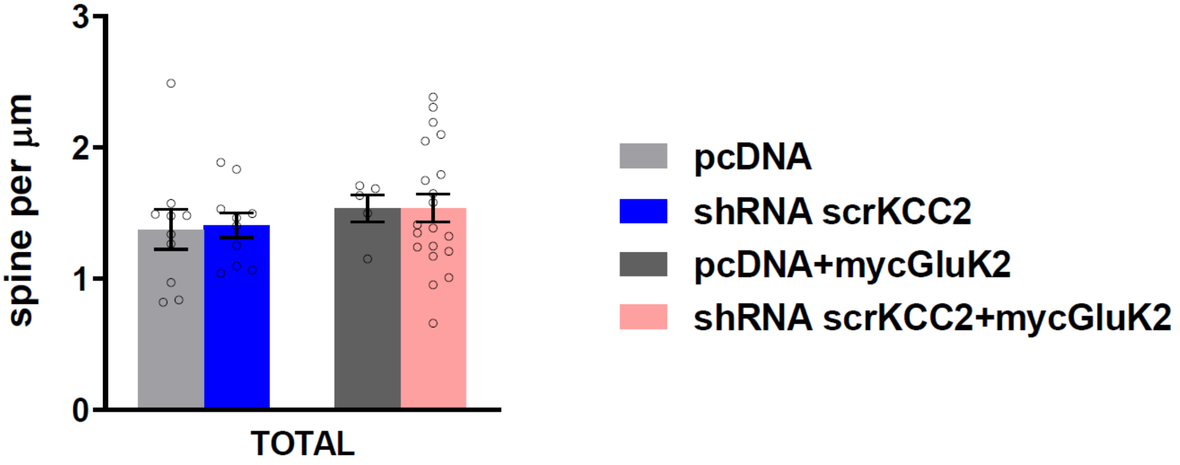

Supplementary Figure 3: Mock shRNA control experiment shows no main impact on dendritic spine density in cultured hippocampal neurons.
